# Semi-automated optimized method to isolate CRISPR/Cas9 edited human pluripotent stem cell clones

**DOI:** 10.1186/s13287-023-03327-2

**Published:** 2023-04-27

**Authors:** Elie Frank, Michel Cailleret, Constantin Nelep, Pascal Fragner, Jérome Polentes, Elise Herardot, Lina El Kassar, Karine Giraud-Triboult, Christelle Monville, Karim Ben M’Barek

**Affiliations:** 1 INSERM U861, I-Stem, AFM, Institute for Stem cell Therapy and Exploration of Monogenic Diseases, 91100 Corbeil-Essonnes, France; 2 U861, I-Stem, AFM, Université Paris-Saclay, Université d’Evry, 91100 Corbeil-Essonnes, France; 3 ALS Automated Lab Solutions GmbH, 07745 Jena, Germany; 4 CECS, Centre d’étude des cellules souches, 91100 Corbeil-Essonnes, France

**Keywords:** CRISPR/Cas9, Gene edition, Human pluripotent stem cell, Clonal isolation

## Abstract

**Background:**

CRISPR/Cas9 editing systems are currently used to generate mutations in a particular gene to mimic a genetic disorder in vitro. Such “disease in a dish” models based on human pluripotent stem cells (hPSCs) offer the opportunity to have access to virtually all cell types of the human body. However, the generation of mutated hPSCs remains fastidious. Current CRISPR/Cas9 editing approaches lead to a mixed cell population containing simultaneously non-edited and a variety of edited cells. These edited hPSCs need therefore to be isolated through manual dilution cloning, which is time-consuming, labor intensive and tedious.

**Methods:**

Following CRISPR/Cas9 edition, we obtained a mixed cell population with various edited cells. We then used a semi-automated robotic platform to isolate single cell-derived clones.

**Results:**

We optimized CRISPR/Cas9 editing to knock out a representative gene and developed a semi-automated method for the clonal isolation of edited hPSCs. This method is faster and more reliable than current manual approaches.

**Conclusions:**

This novel method of hPSC clonal isolation will greatly improve and upscale the generation of edited hPSCs required for downstream applications including disease modeling and drug screening.

**Graphical Abstract:**

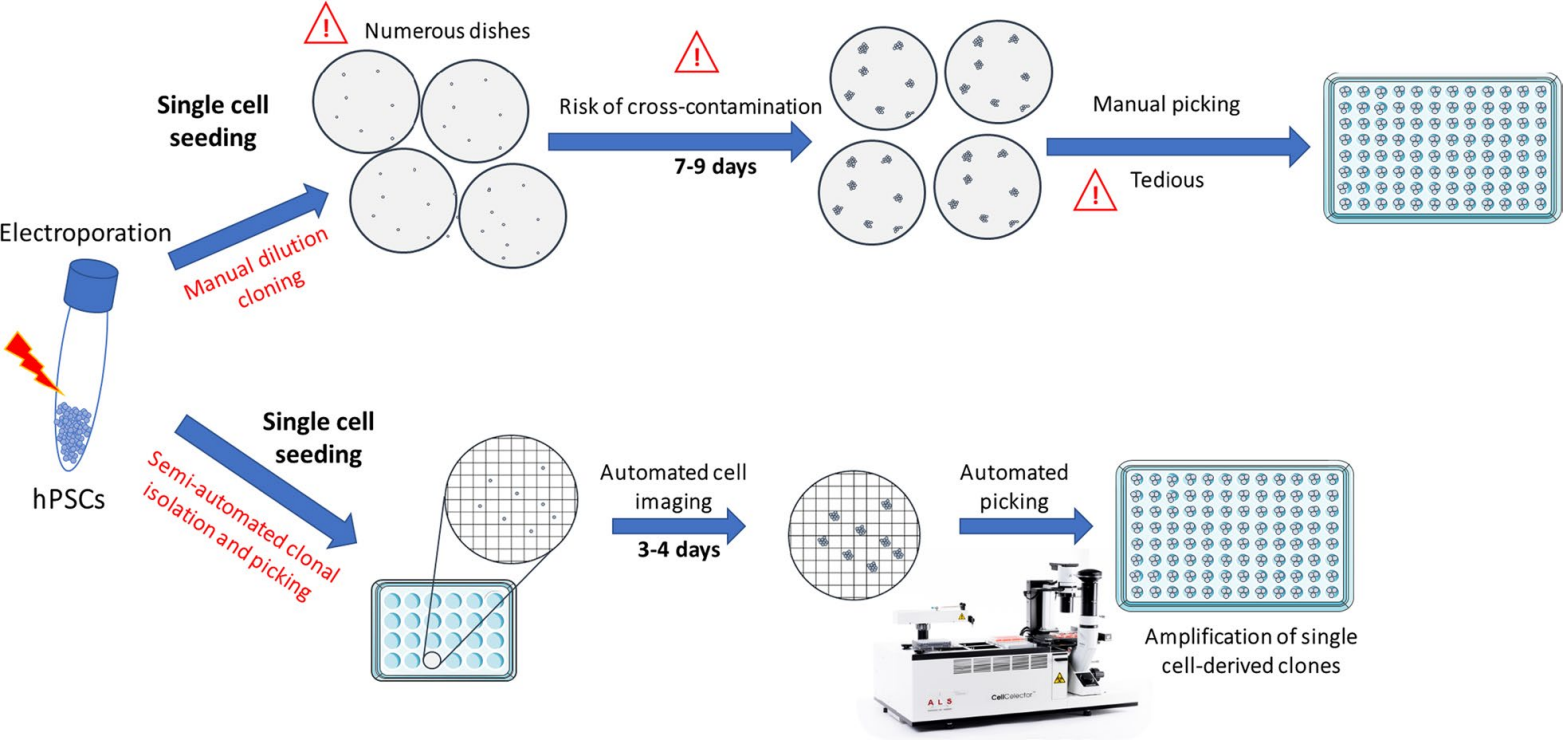

**Supplementary Information:**

The online version contains supplementary material available at 10.1186/s13287-023-03327-2.

## Introduction

Disease modeling of human pathologies in vitro using human pluripotent stem cells (hPSCs) is exponentially increasing in the scientific community [15, 22]. While retaining self-renewal capacity allowing large-scale expansion, these cells hold the potential to give rise to any differentiated cell type of the adult body if appropriate signaling cues are provided [25]. Thus, hPSCs allow obtaining adult differentiated cells and tissues that would be difficult to obtain otherwise. Indeed, sampling specific cells or tissues from a patient can be traumatic or impossible to perform without taking the risk to cause irreparable damages (e.g., brain areas and retina). Moreover, some organs contain non-proliferating cells that cannot be cultured and amplified after sampling (e.g., neurons).

A variety of differentiation protocols from hPSCs in vitro were already developed to generate different adult cell types such as retinal pigment epithelial cells [20], photoreceptors [10], keratinocytes [13] or cardiomyocytes [29]. A drawback of hPSC disease modeling is the need for several genetic disease patient and control cell lines to decipher molecular pathological mechanisms of a specific gene. Gene edition technology overcomes these obstacles and isogenic hPSC models have been generated through gene edition to study the contribution of a specific mutation to a pathology in a controlled genetic background.

Clustered regularly interspaced short palindromic repeats (CRISPR) and its CRISPR-associated protein Cas9 is a bacterial mechanism of adaptive immunity that can be used to edit DNA within living cells [1, 16]. Cas9 protein forms a complex with a selected guide RNA to induce a double-stranded break at a targeted locus. DNA repair processes engender a variety of insertions and deletions (indels) at the DNA cleavage site. These indels can result in the knockout of a selected gene. Alternatively, when a homologous DNA template is added concomitantly with CRISPR/Cas9-guide RNA complexes, DNA repair through homology-directed repair elicits a desired modification contained in the DNA template to correct a gene mutation or introduce a novel sequence. More recently, Komor et al. developed a base editor combining catalytically impaired Cas protein associated with a deaminase [18]. Such system allows precise base substitutions to mimic or correct point mutations associated to a pathology. Although recent updates of the CRISPR/Cas9 edition system have been published [23], this method is still limited by its maximum efficiency rate [21, 28]. Indeed, this rate can be variable and depends on guide RNA [23], on the type of Cas9 [17] as well as its delivery method [24, 28]. Therefore, following the gene edition of a cell population, isolation of clones is required to separate non-edited and different types of edited cells [7]. The standard approach consists of manual selection and harvesting of clones in culture dishes plated with very low cell densities (dilution cloning) [12]. Such approach is time-consuming and labor intensive. In addition, there is a risk that clones could be a mixture of more than one cell. A risk that can be mitigated by two successive dilution clonings.

To overcome these limitations, we developed a novel approach to isolate single cells from a mixed cell population following CRISPR/Cas9 gene edition and to pick colonies derived from these isolated single cells. Our method relies on a semi-automated robotic platform (CellCelector™), which combines an epifluorescence microscope setup associated with a dedicated software allowing automated single cell identification and clonal follow-up, and a robotic arm to collect identified clones. We first showed that this semi-automated method is reliable to isolate single cell-derived clones without mixing cells during cell culture using dedicated Cellcelector nanowell culture plates. Then, we demonstrated how this method could be applied for a CRISPR/Cas9 gene editing experiment in hPSCs in order to model a genetic disorder: the Alström Syndrome (AS). This novel method is robust, easier and faster than standard dilution cloning and may be up scaled to generate various gene edition experiments at the same time.

## Methods

### hPSCs culture

Wild-type hiPSCs PC056c2 were derived by Phenocell (Grasse, France) from human primary fibroblasts using Sendai vectors [2] and cultured with StemMACS iPS-Brew XF medium (Miltenyi Biotec) in vitronectin (Gibco-coated dishes). Culture medium was changed three times a week. Cells were passaged following gentle harvesting with EDTA 0.25 mM (Invitrogen every 5–7 days). For CRISPR/Cas9 editing experiments, hiPSCs were pretreated with a rock inhibitor (Y-27632; Tocris) at least one-hour prior transfection and harvested with an enzymatic treatment (Stempro Accutase cell dissociation reagent; Life technologies). Following amplification of single cell-derived clones, cells were banked using CryoStor CS10 (Sigma-Aldrich).

### CRISPR/Cas9 gene editing

For p.Glu192fs mutation, guide RNA (gRNA) was selected based on CRISPOR.org predictions [8]. Twenty possible gRNAs were proposed of which 18 gRNAs with a required MIT specificity score superior to 50. Of these 18 gRNAs, we selected one with both the higher Lindel score (prediction probability of frameshift) and the higher Doench’16 score (predicted cleavage efficiency). One hundred thirty-seven off-targets were predicted with up to four mismatches with the original guide sequence. Of these, the top 15 off-targets were not localized into an exon. Integrated DNA Technologies (Iowa, USA) synthesized selected gRNA. hiPSCs were plated in 24-well plates at 80–120,000 cells per well. Lipid-based transfection was performed the next day with 15 µM of ribonucleoprotein (RNP) complexes (gRNA, SpCas9 protein), according to the manufacturer’s protocol (Lipofectamine Stem Transfection Reagent, ThermoFisher Scientific). SpCas9 purified protein is a gift from Jean-Paul Concordet (MNHN-CNRS UMR 7196/INSERM U1154).

After enzymatic dissociation with Stempro Accutase (Life technologies), 150,000 hiPSCs in 7.5 µL of resuspension buffer (Neon Transfection Kit, Invitrogen) were mixed with 7.5 µL of RNP complexes. The mix was then electroporated at 1200 V/20 ms/2 pulses using 10 µL Neon Tips. For limiting dilution cloning experiments, the cell suspension was plated in several 100-mm culture dishes at different concentrations (1000/2000/5000/10,000 cells/dish). For semi-automated cell picking, cells were seeded in 6-well plates or directly seeded following electroporation in a vitronectin-coated nanowell plate at a density of 3000 cells per well.

For the base editing strategy, different pathogenic nonsense mutations were identified within *ALMS1* using the ClinVar database (NCBI). Among the identified mutations, we selected the p.Gln3613Ter mutation as it consists in a C → T nonsense mutation. A gRNA targeting this position was then designed using CRISPR RGEN Tools and synthesized by Integrated DNA Technologies (Iowa, USA).

After enzymatic dissociation with Stempro Accutase (Life technologies), 150,000 hiPSCs in 10 µL of resuspension buffer (Neon Transfection Kit, Invitrogen) were mixed with 10 µL of RNA AncBE4max (pCMV_AncBE4max_P2A_GFP was a gift from David Liu (Addgene plasmid # 112100; http://n2t.net/addgene:112100; RRID: Addgene_112100)) and 2.5 pmol gRNA. The mix was then electroporated using the same protocol. For semi-automated cell picking, cells were seeded in a vitronectin-coated nanowell plate at a density of 3,000 cells per well.

### Sequence analysis

Genomic DNA was extracted with QuickExtract DNA extraction solution (Lucigen) according to the manufacturer’s instructions. PCR amplification corresponding to the edited DNA sequence (p.Glu192fs: forward primer: TATACACTGGCAGCGGCG; reverse primer: TCTGTAGAGACCTACTCAGAGGG; p.Gln3613Ter: forward primer: CCCGTACACCTACCAAGTGA; reverse primer: CATCATCGAGCTGGGAGGTC) was subjected to Sanger sequencing (Genewiz, Germany). To evaluate edition rates, decomposition of quantitative sequence trace data was based on TIDE [4]. Sequence analysis was performed using Snapgene software (Insightful Science).

### CellCelector clonal isolation

24-well nanowell plates (ALS, Germany) were coated with vitronectin (Gibco) and centrifugated for 5 min at 1258 g (to eliminate micro-bubbles) prior to incubation at 37 °C for 1 h. Approximately 3000 cells were seeded per well (4300 square-shaped nanowells in total in a well of a 24-well plate). Seeded cells are randomly distributed in nanowells following Poisson distribution resulting in ~ 30% of nanowells occupied with single cells.

After cell seeding, plates were centrifuged again for 3 min at 100 g to allow all cells to be captured in nanowells. Nanowells with seeded cells were automatically scanned on the CellCelector system (ALS, Germany) on D0 just after cell seeding. Identification of single cell nanowells on D0 was performed using a specific image analysis algorithm using CellCelector software (v3.1) including gating with single cell size and sphericity. At time of picking on D3-5, the nanowell plate was scanned again and the system automatically selected single cell-derived colonies based on their outgrowth. Selected single cell-derived colonies were detached with Stempro Accutase (Life technologies) just prior to picking run. Single-use CellCelector glass capillaries (ALS, Germany) with internal diameter of 80 µm with aspiration volume of 0.5 µL were used for picking. The tip of the capillary was calibrated at the height of approximately 80 µm from the bottom of nanowells. Colonies were deposited in a vitronectin-coated 96-well plate with 200 µL of StemMACS iPS-Brew XF medium (Miltenyi Biotec) containing rock inhibitor. Cell growth was followed using INCUCYTE S3 (Sartorius).

### Flow cytometry

Cells were dissociated with TrypLE (ThermoFisher) and stained with TRA1-81-AF647 (Biolegend) or SSEA-4-PE (Miltenyi Biotec) and corresponding isotypes from the same suppliers. For intracellular markers, cells were fixed with 4% PFA following dissociation. Cells were treated with PBS 0.1% tritonX100 and then incubated overnight with OCT3/4 (Santa Cruz, sc-5279, 1:100) and LIN28A (R&D SYSTEMS, AF3757, 1:200). The day after, cells were washed and incubated with corresponding secondary antibodies (Invitrogen). Acquisitions were done with a MACSQuant analyzer (Miltenyi Biotec) and data were analyzed using the FlowJo software.

### Immunofluorescence

After fixation with 4% PFA for 15 min at room temperature, cells were incubated overnight at 4 °C with the following primary antibodies: OCT3/4 (Santa Cruz, sc-5279, 1:100), LIN28A (R&D SYSTEMS, AF3757, 1:200), Pericentrin (Abcam, ab28144, 1:200) and ALMS1 (Abcam, ab84892, 1:200). Appropriate Alexa fluorescent secondary antibodies (Invitrogen) and DAPI were added for 1 h. Image acquisitions were performed on a Zeiss LSM880-Airyscan Confocal Microscope driven by the Zeiss Zen black software.

### Single-nucleotide polymorphism (SNP) analysis

High-quality genomic DNA was obtained with Nucleospin Tissue kit (Macherey Nagel) according to manufacturer instruction. gDNA hybridization was achieved on Infinium Core-24v1-2 BeadChip (Illumina). Data were analyzed with GenomeStudio v2.0.5 software (Illumina).

### Multiplex fluorescence in situ hybridization (mFISH) karyotype analysis

Cells were blocked in metaphase with colchicine (Eurobio) for 90 min and then they were detached with trypsin. After centrifugation, the supernatant was removed and cell pellet was suspended and warmed in a hypotonic solution for several minutes and then fixed with a Carnoy fixative. Drops of the cell suspension were spread on glass slides and let to dry. mFISH 24Xcite probe (MetaSystems) and ProLong Gold Antifade Mountant with DAPI (Thermo Fisher Scientific) were used for mFISH staining. Seventy metaphases were acquired with Metafer MetaSystems software coupled to an AxioImager Zeiss Z2 microscope furnished with a camera cool cube and 10X and 63X objectives. Images were analyzed with Isis software (MetaSystems).

## Results

### Accurate algorithmic identification of square-shaped nanowells

**Fig. 1:**
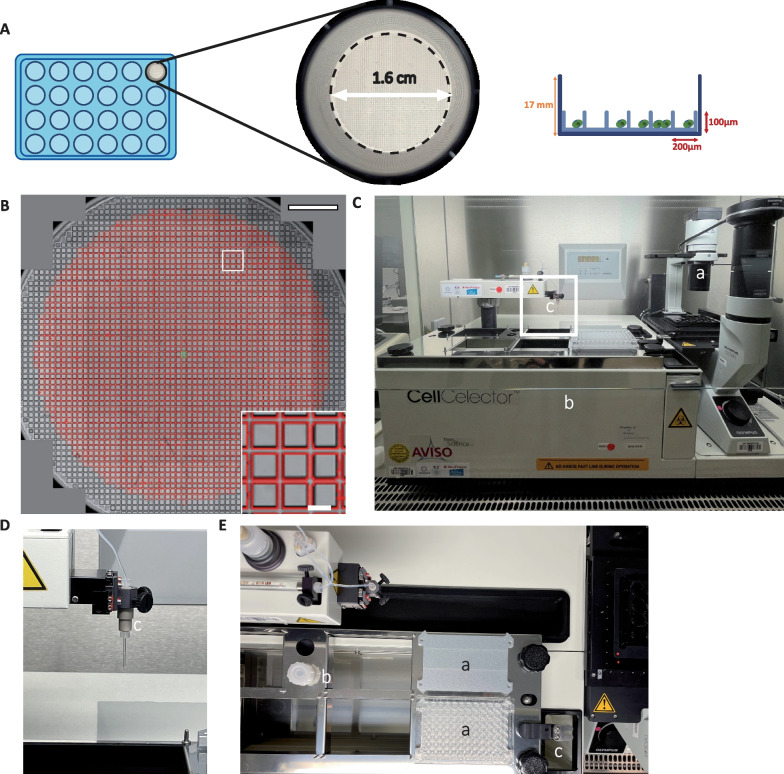
Algorithm identification of square-shaped nanowells. **A** Illustration of a CellCelector 24-well nanowell plate. Each nanowell forms a square (200 × 200 µm) delimited by 100 µm height walls. Well diameter = 1.6 cm. **B** Automated segmentation of nanowells in the automatically stitch whole well image. In each representative well, the software identifies by segmentation 2263 square-shaped nanowells (1 mm peripheral region of the well is not analyzed). Each identified nanowell is surrounded by red lines and assigned a unique ID for traceability through the experiment. Insert corresponds to magnified area. Scale bar = 2 mm and for magnification = 200 µm. **C.** Overview of the CellCelector™ system: (a) inverted fluorescence microscope and motorized stage; (b) platform to receive destination plates and (c) cell picking robotized arm with high-precision glass capillary (magnification in **D**). **E** Upper view of the robotic system: the platform is composed of dedicated areas: (a) destination plates, (b) sterilization tank and (c) waste container

Current hPSC culture methods involve passaging as small clumps/aggregates to prevent cell death or cellular stress and to prevent the risk of genetic alterations [11]. Following gene edition, hPSCs need to be isolated as single cells for further clonal isolation and genotype selection. To minimize space taken by many culture dishes seeded at low cell dilution, we used one well of a 24-well plate (Fig. 1A). This well is micro-structured with square nanowells (volume of approximately 4 nL). In that context, the presence of nanowells allows to increase cell density in the well, while keeping single cells efficiently separated from each other by nanowell walls, yet covered by the same medium. This enables to co-culture in adherence cells in the same large macro-well while maintaining their monoclonality. Endogenous factors secreted by hPSCs that favor pluripotency and support clonal proliferation may be more concentrated, reducing therefore cellular stress [14]. We evaluated first the ability of the software algorithm to isolate individual nanowells. The algorithm was able to recognize and segment 2031 ± 34 individual squares in a well of a 24-well plate (Fig. 1B). Thus, this number corresponds to theoretical single cells that can grow separately in each individual identified nanowells to form clones that can be collected.

### Single cells grow in adherence inside nanowells and form colonies pickable using a robotized arm

**Fig. 2:**
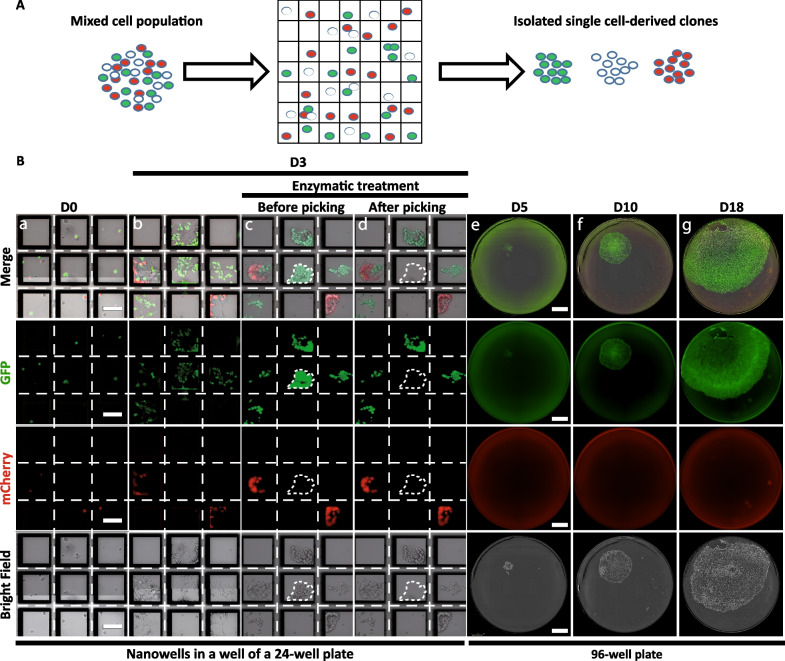
Automated isolation of single cell-derived clones in nanowells. **A** Schematic representation of the automated isolation of single cell-derived hPSC clones. A mixed population of label-free, mCherry- and GFP-labeled hiPSCs are mixed (1:1:1) prior single cell seeding into nanowell plates and automated clone picking. **B** Representative images of label-free hiPSCs, GFP-hiPSCs and mCherry-hiPSCs seeded as single cells at day 0 (D0; a), at 3 days (D3) of culture within nanowells (b), after enzymatic treatment to harvest clones (c) and after picking by the automated arm of the CellCelector™ (d). The selected square-shaped micro-pattern for picking is at the center of the image. The picked single cell-derived clone is highlighted with dotted lines in panels (c) and (d). Following picking, colonies are deposited into a 96-well plate where their growth is followed by imaging at D5, D10 and D18 (e, f, g). Scale bar in 24-well plate images = 100 µm; scale bar in 96-well plate images = 500 µm

To identify single cells in nanowells and collect single cell-derived clones, we used the CellCelector™ automated system composed of a standard inverted fluorescent microscope equipped with a camera, a motorized stage and a robotized arm with a mounted thin glass capillary (Fig. 1C–E). This system is associated with a software that allows precise detection of single cells in nanowells and selection of single cell-derived colonies. In order to validate the ability of this system to recognize, select and pick hPSC clones, we generated two knock-in hPSC lines with constitutive expression of GFP or mCherry, the transgenes being inserted into adeno-associated virus integration site 1 (*AAVS1)* locus (Additional file 1: Fig. S1). We then mixed in equal proportions label-free, GFP- and mCherry-expressing hPSCs before seeding them as single cells into square-shaped nanowells (Fig. 2A). After cell seeding on day zero (D0), the integrated imaging setup scanned the seeded wells to automatically identify nanowells containing only one cell, thus providing a robust and documented image-based monoclonality proof (Fig. 2B). On day 3 (D3), a second scan was performed to automatically identify grown hPSC colonies derived from single cells according to the spatial position of each nanowell in the well. An enzymatic treatment with Accutase® was applied to gently harvest colonies that grew in adherence. The robotic arm was then used to collect selected single cell-derived clones and to transfer them into a new 96-well culture plate (Additional file 2: Movie 1). Following a few additional days of proliferation, cells were imaged and analyzed for single cell clonality. We showed that this semi-automated system selectively picked colonies without contamination from neighboring cells (expressing another fluorescent protein or without fluorescence) and/or significant loss of cell survival during the collection process.

### No cross-contamination following sequential cell colony picking using the same capillary

**Fig. 3:**
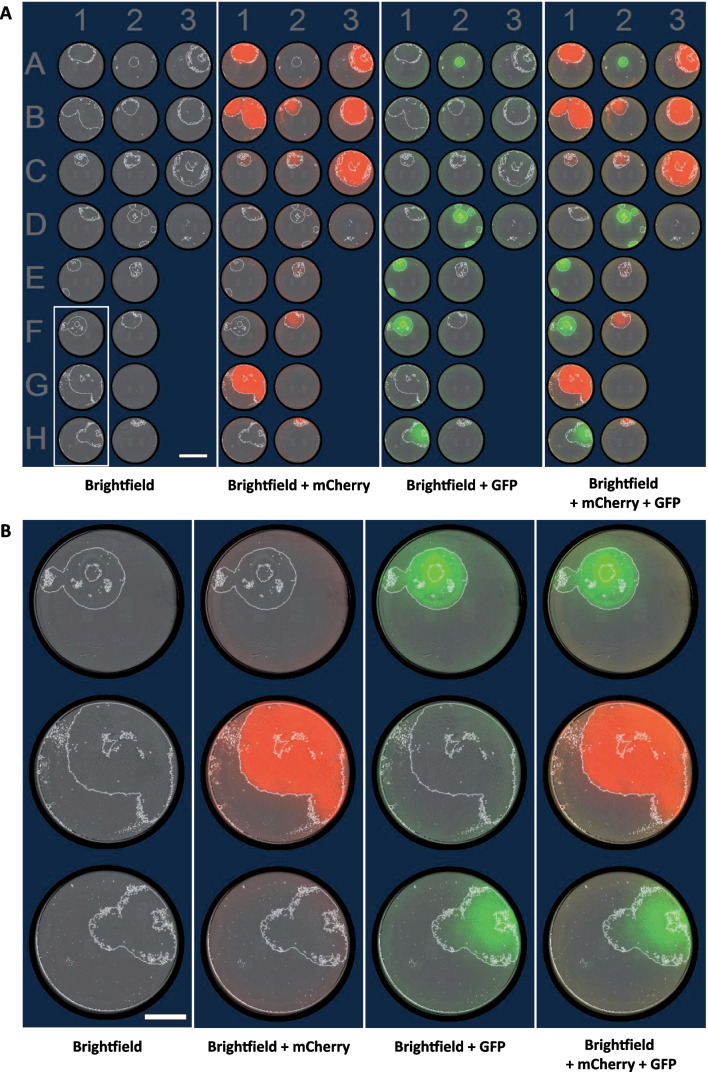
Sequential automated picking of single cell-derived clones does not engender cross-contamination. **A** Representative images of 96-well plate containing single cell-derived colonies 11 days after automated cell picking. The picking order started from A1 to H1, A2 to H2 and finally A3 to D3. Colonies are surrounded with white lines. Scale bar = 4 cm. **B** Magnification of wells F1, G1 and H1. Picking order started from F1 to H1. Scale bar = 2 cm

As the glass capillary is not changed between each individual picks, we evaluated if using the same capillary to pick several single cell-derived colonies sequentially would result in any cross-contamination (Fig. 3). We collected single cell-derived colonies of different single labeling (label-free, GFP or mCherry fluorescence). We did not observe fluorescent color mixing between different consecutive single cell-derived clone pickings and amplification. Thus, we showed that sequential picking of different colonies did not induce cross-contamination.

### CRISPR/Cas9 gene edited hPSCs are efficiently isolated using a semi-automated method

**Fig. 4:**
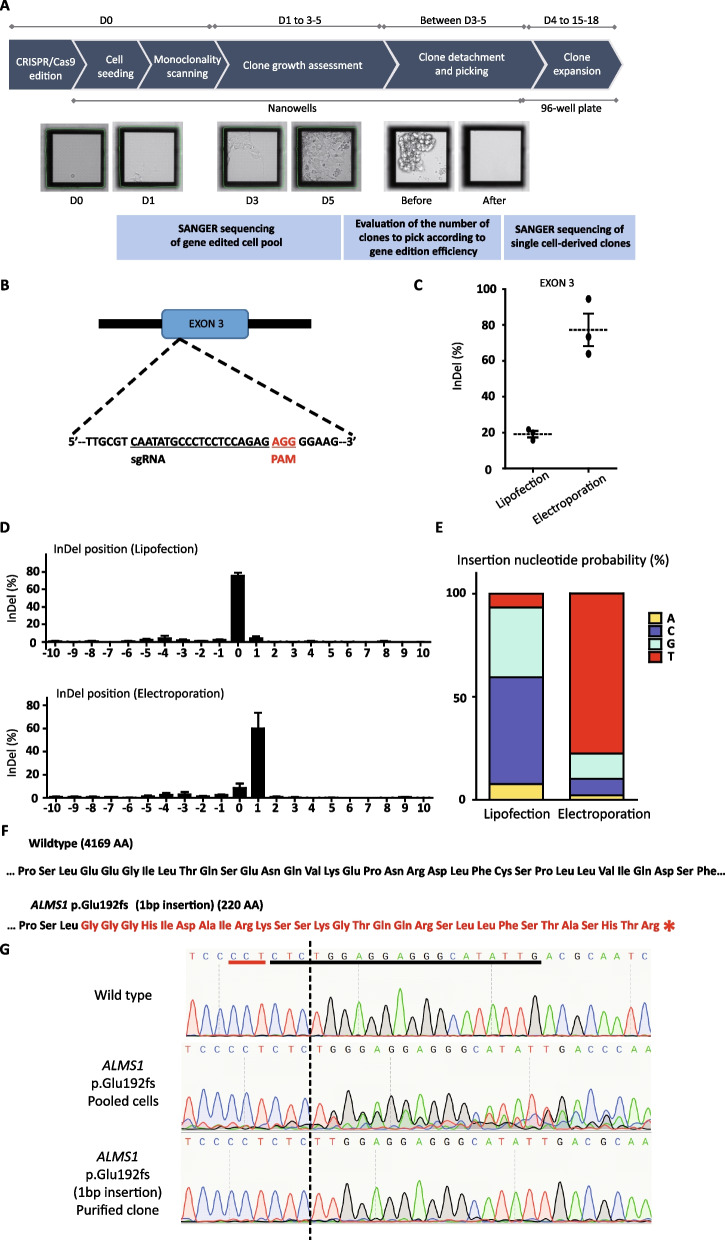
Induction of a frameshift mutation in ALMS1 with CRISPR/Cas9 gene editing. **A** Workflow from gene edition to single cell-derived clone semi-automated isolation and amplification. **B** Schematic representation of the targeted area (exon 3 of ALMS1 gene) by CRISPR/ Cas9 with the selected gRNA. PAM = protospacer adjacent motif. **C** Quantification of the percentage of indels following CRISPR/Cas9 according to the mode of transfection. Electroporation method leads to a higher rate of indel events within the cell population compared to lipofection. **D** Evaluation of the percentage of indels according to the nucleotide position. (0 corresponds to the Cas9 cleavage site). **E** Evaluation of the probability of nucleotide insertion according to the type of transfection based on sequencing results**. F** Comparison of the predicted amino acid sequence of ALMS1 protein in WT cells and KO purified clones (with + 1 bp insertion). Following gene edition, the sequence of amino acids is modified (red letters) and a STOP codon appears quickly downstream of the insertion site (red star). **G** Sequences of the site of gene edition before CRISPR/Cas9 editing (wild type); following edition in a representative edited cell population (knock out pooled cells), and in a representative single cell-derived clone using the semi-automated method. The gRNA is underlined in black and the PAM motif in red. The dotted black line corresponds to the Cas9 cleavage site. Mean of 3 independent experiments

As a proof of concept, we selected the *ALMS1* gene for CRISPR/Cas9 editing to create a Knockout model of AS. *ALMS1* is composed of 23 exons and codes for a protein of 4169 amino acids [9]. We targeted the exon 3 (near the 5’ end of the coding region) to identify potential guide RNAs for CRISPR/Cas9 edition for which indels may cause a major deletion into the resulting protein. Based on in silico analysis, we selected a guide RNA out of 20 with best-predicted performances (Fig. 4B). We evaluated the efficiency of gene edition using either electroporation or lipofection approaches using this same guide RNA. Sequence analysis of edited cell pools revealed that electroporation engendered significantly more indels than lipofection (77% versus 19% respectively; Fig. 4C). Following sequence trace decomposition of SANGER sequencing, we estimated the frequency and types of indels. Lipofection induced a diversity of indels at low frequencies whereas electroporation with the same guide engendered mostly one nucleotide insertion (thymine, Fig. 4D, E). This last type of edition *ALMS1* p.Glu192fs was predicted to induce a frameshift that can lead to a premature stop codon (resulting in a shortened protein of 220 amino acids; Fig. 4F). We then dissociated this edited hPSCs population as single cell and seeded the cells in square-shaped nanowells. Single cell-derived colonies were identified by the software algorithm and picked using the CellCeletor™ robot. Sequence analysis of single cell-derived clones allowed to detect pure clones bearing the desired mutation (+ 1 insertion; Fig. 4G).

### CRISPR/Cas9 gene edited clones maintained their pluripotency and genetic integrity following isolation and banking

**Fig. 5:**
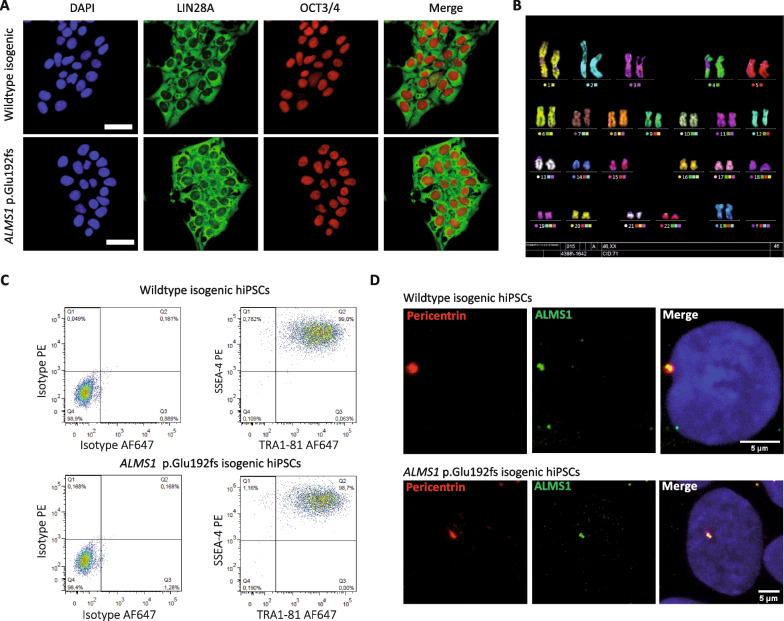
Single cell-derived clones CRISPR/Cas9 edited remain pluripotent and without genomic aberrations. **A** Immunofluorescence images of hPSC colonies (WT before selection and p.Glu192fs purified clone) stained for pluripotency markers OCT3/4 (red) and LIN28a (green). Scale bar = 50 µm. **B** Karyotype of a p.Glu192fs purified clone. **C** Flow cytometry analysis of pluripotency markers TRA1-81 and SSEA-4 on WT before selection (upper panel) and p.Glu192fs hPSCs (lower panel). **D** Immunofluorescence images of hPSCs stained for Pericentrin (red) and ALMS1 (green). Scale bar = 5 µm

Single cell-derived hPSC clones of interest were amplified and banked. We then evaluated the pluripotency state and genomic integrity of hPSCs following gene edition, semi-automated clonal isolation and cell banking of amplified clones. Using immunofluorescence for pluripotency markers, we showed that isolated clones maintained a pluripotency state as evaluated by the expression of OCT3/4 and LIN28 (Fig. 5A). Flow cytometry analysis for cell surface markers Tra1-81 and SSEA-4 confirmed also pluripotency (Fig. 5C). Genomic integrity was evaluated by karyotype and single-nucleotide polymorphism (SNP) comparison between parental hPSCs and edited clones following amplification and banking (Fig. 5B and Additional file 3: Fig. S2). We did not find neither chromosomal aberrations nor genetic variations between parental and the majority of edited cell lines, suggesting that genomic integrity was preserved during the process. We next evaluated by immunofluorescence the presence of ALMS1. The protein was present at the centrosome (co-expression with Pericentrin) in both WT and edited cells (Fig. 5D). Thus, the in silico prediction was not verified with this particular mutation as the antibody epitope is localized after the mutation.

**Fig. 6:**
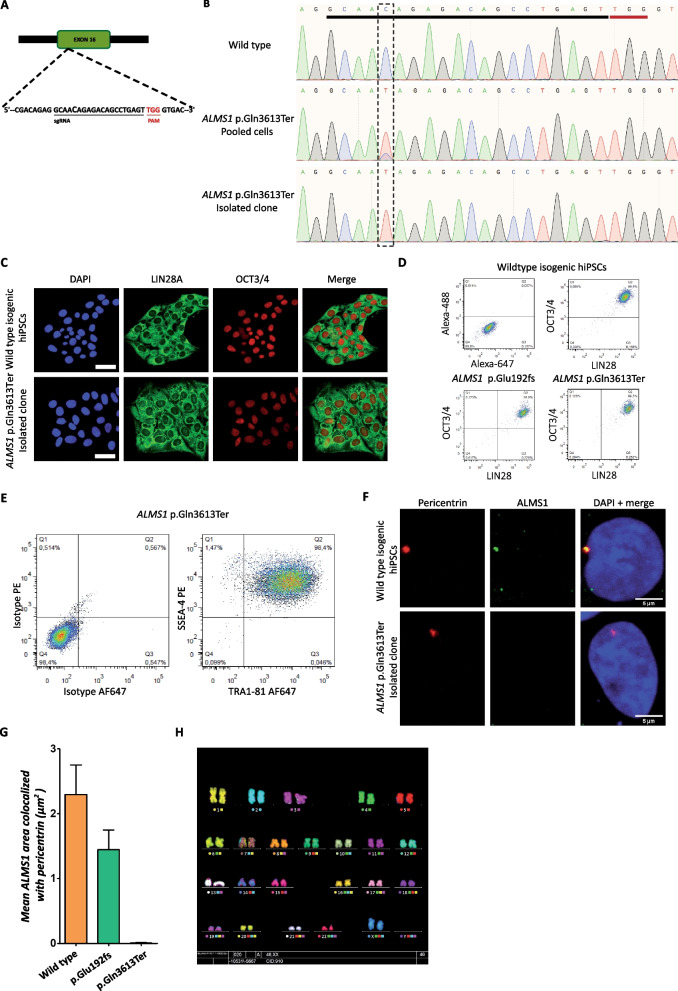
CRISPR/Cas9 base edition and single cell-derived clone characterization. **A** Schematic representation of the region (exon 16 in ALMS1 gene) targeted by CRISPR/Cas9 with the selected gRNA. **B** SANGER sequences of the site of edition before CRISPR/Cas9 base edition (WT before selection; upper panel), following edition in a representative edited cell population (pooled cells; middle panel), and in a representative single cell-derived clone using the semi-automated method (lower panel). The gRNA is underlined in black and the PAM motif in red. The dotted rectangle corresponds to the position targeted for base editing. **C** Immunofluorescence assay using pluripotency markers (OCT3/4 and LIN28A) on WT before selection and p.Gln3613Ter purified hPSC clone (lower panel). Scale bar = 50 µm. **D**, **E** Evaluation of pluripotency of p.Gln3613Ter and p.Glu192fs hPSCs by flow cytometry (pluripotency markers OCT3/4 and LIN28A (**D**) and TRA1-81 and SSEA-4 (**E**)). **F** Immunofluorescence assay showing the expression of Pericentrin (red) and ALMS1 (green) on WT (upper panel) and p.Gln3613Ter purified hPSC clone (lower). Scale bar = 5 µm. **G** Quantification of ALMS1 area (µm^2^) co-localized with Pericentrin according to the genotype (wild type, p.Glu192fs, p.Gln3613Ter). **H** Karyotype of the p.Gln3613Ter purified hPSC clone

We also applied this semi-automated method to isolated CRISPR/Cas9 gene edited clones obtained with a base editing approach. We identified a guide RNA to induce a base substitution at a key locus that engenders a premature termination codon (*ALMS1* p.Gln3613Ter) (Fig. 6A). This point mutation is a phenocopy of an already identified pathological mutation (CLINVAR NCBI website). We edited cells using an electroporation approach. Sequence trace decomposition of SANGER sequencing revealed a base editing rate of 78% (Fig. 6B). After edition, single cells were seeded into nanowells and single cell-derived colonies were collected 3–4 days later. Colonies were banked and analyzed for pluripotency markers (immunofluorescence and flow cytometry; Fig. 6C–E), and genetic integrity (karyotype and SNP comparison to original hiPSCs; Fig. 6H). Base editing and clonal isolation did not affect cell quality. Immunofluorescence for ALMS1 revealed the absence of the protein in edited cell confirming the protein loss (Fig. 6F, G).

## Discussion

Herein, we developed a semi-automated method to isolate single cell-derived hPSC clones cultured in adherence. Dissociated cells were seeded into square-shaped nanowells inside wells of a 24-well plate. We identified single cells and followed their proliferation for several days with microscopic image recordings to ensure the traceability of clonality. Colonies were then picked up using a robotic arm and subcultured into new culture plates. We successfully implemented this method to isolate single cell-derived clones from CRISPR/Cas9 gene edited hPSCs. Finally, we developed a simple workflow from hPSC gene editing to clonal isolation and cell banking (Fig. 4A).

Current standard clonal isolation of hPSCs involves dissociation as single cells and manual dilution at very low cell density in numerous culture dishes. This approach is tedious and labor-intensive. Our semi-automated clonal isolation method is faster and more robust than standard dilution cloning. Firstly, we reduce the burden of handling numerous culture dishes by using only one nanowell-containing well of a 24-well plate for each type of cell line/experiment. Indeed, beside the method used to isolate single cells, all cells will not recover and form colonies: an estimation of 1% of hPSCs may survive [27]. This survival rate is improved up to 27% with the addition of rock inhibitor [27]. Thus, the cell culture in micro-pattern nanowells allows the selection and amplification of potentially 2000 collectable single cells in the same well of a 24-well plate (corresponding to twenty 96-well plates). Our method does not improve survival rates but increases the number of cells followed (throughput) to diminish the effects of these losses. Using this approach, only cells that are proliferative, are subsequently collected and subcultured into new plates. Our strategy is therefore different from cell dispensers or cell sorters that dispatch single cells in wells of 96-well plates or equivalent [5, 26].

Secondly, nanowells allow the separation of one cell from another and a microscopic image allows the follow-up of each single cell growing as a new colony (traceability of clonality). At the opposite, traceability of cultured cells is limited when cells are dispatched with dilution cloning without using adapted separating wells: hPSCs are susceptible to migrate, proliferate and form colonies that could further split into two other colonies or could fuse to form mixed colonies [6]. This risk of non-clonal colony formation is mitigated by low-density cell seeding [19]. Some protocols even recommend two sequential manual dilution clonings to ensure single cell-derived colonies [3].

Thirdly, identified single cell-derived colonies are picked and subcultured based on acquired microscopy images with a robotic arm. This automated picking is an advantage as it suppresses human manipulations and associated risks (fungi/bacterial contamination, picking errors).

Efficiency of gene editing using CRISPR/Cas9 or other editing strategies is variable and depends on numerous factors (*e.g.,* gene locus, Cas9 variant, gRNA, etc.). When editing efficiency is low, a high number of clones need to be isolated to obtain the few clones that are homozygotes for the desired mutation [5]. Thus, some protocols combined CRISPR/Cas9 editing with fluorescence-activated cell sorting to isolate single cell-derived clones into wells of 96-well plates [5]. This engenders the handling of an important number of plates. In addition, in this context, cell sorting is stressful and cell survival is low (about 29–47% of sorted cells will form colonies). Our semi-automatic method is particularly adapted to such experiments with the combination of nanowell cultures and automated picking. We developed a workflow from gene edition and seeding on the same day in nanowells to the automated picking and subculture into new plates. In particular, the time interval between cell seeding and picking (3–4 days) allows to analyze the pool of edited cells to estimate edition efficiency and adapt accordingly the number of single cell-derived clones to collect. Of note, additional testing may be required to fully demonstrate pluripotency of the cells that have undergone the whole selection process (ability to differentiate into the three germ layers).

Finally, the workflow that we developed is compatible with upscaling and several gene edition experiments can be performed at the same time in different wells of 24-well plate (each well being treated independently). Following amplification of single cell-derived hPSC clones, these cells can be used for downstream applications including identification of molecular mechanisms or high throughput drug screenings.

## Conclusions

We developed a semi-automated method to isolate reliably single cell-derived clones following gene edition with reduced manual handling, reduced number of culture dishes and increased traceability. This method is completely different from other automated methods that essentially rely on cell dispensers’ strategies following gene edition into 96-well plates. This new semi-automated method will be useful for the hPSC research community allowing efficient generation of hPSCs modeling genetic disorders or for the study of gene functions.

## Supplementary Information

**Additional file 1: Fig. S1.** Upper panel: Design of CRISPR/Cas9 edition into AAVS1 locus and donor DNA insertion to generate hiPSCs with constitutive expression of GFP and mCherry. Middle panel: Flow cytometry analysis of pluripotency markers SSEA-4 and TRA1-81 of edited hPSCs. Lower panel: images of edited hiPSCs with constitutive expression of GFP or mCherry.

**Additional file 2: Movie 1.** Automated picking of single cell-derived hiPSC clones and seeding into new plates.

**Additional file 3: Fig. S2.** A Single-nucleotide polymorphismcomparative analysis of parental cells and a representative edited single cell-derived hiPSC clone. B Table recapitulating the comparative analysis of original hiPSCs and clones derived with ALMS1 p.Glu192fs and ALMS1 p.Gln3613Ter mutations. C Number of clones analyzed for SNPs according to the CRISPR/Cas9 experiment.

## Data Availability

Data and cell materials presented in this study are available upon request. SANGER sequences and SNP datasets are available at Mendeley data (10.17632/s94y6mw87f.1).

## Abbreviations

hPSCs: Human pluripotent stem cells
CRISPR: Clustered regularly interspaced short palindromic repeats
Indels: Insertions and deletions
DNA: Deoxyribonucleic acid
RNA: Ribonucleic acid
AS: Alström syndrome
gRNA: Guide RNA
RNP: Ribonucleoprotein
SNP: Single-nucleotide polymorphism
mFISH: Multiplex fluorescence in situ hybridization

## References

[1] Asmamaw M, Zawdie B (2021). Mechanism and applications of CRISPR/Cas-9-mediated genome editing. Biol Targets Ther.

[2] Barrault L, Gide J, Qing T, Lesueur L, Tost J, Denis JA, Cailleret M, Aubry L, Peschanski M, Martinat C (2019). Expression of miRNAs from the imprinted DLK1/DIO3 locus signals the osteogenic potential of human pluripotent stem cells. Cells.

[3] Bower OJ, McCarthy A, Lea RA, Alanis-Lobato G, Zohren J, Gerri C, Turner JMA, Niakan KK (2021). Generating CRISPR-Cas9-mediated null mutations and screening targeting efficiency in human pluripotent stem cells. Curr Protoc.

[4] Brinkman EK, Chen T, Amendola M, van Steensel B (2014). Easy quantitative assessment of genome editing by sequence trace decomposition. Nucleic Acids Res.

[5] Caillaud A, Leveque A, Thedrez A, Girardeau A, Canac R, Bray L, Baudic M, Barc J, Gaborit N, Lamirault G (2022). FACS-assisted CRISPR-Cas9 genome editing of human induced pluripotent stem cells. STAR Protoc.

[6] Chang J, Kim MH, Agung E, Senda S, Kino-Oka M (2019). Effect of migratory behaviors on human induced pluripotent stem cell colony formation on different extracellular matrix proteins. Regen Ther.

[7] Choi DK, Kim YK, HoonYu J, Min SH, Park SW (2021). Genome editing of hPSCs: Recent progress in hPSC-based disease modeling for understanding disease mechanisms. Prog Mol Biol Transl Sci.

[8] Concordet JP, Haeussler M (2018). CRISPOR: intuitive guide selection for CRISPR/Cas9 genome editing experiments and screens. Nucleic Acids Res.

[9] Eintracht J, Forsythe E, May-Simera H, Moosajee M (2021). Translational readthrough of ciliopathy genes BBS2 and ALMS1 restores protein, ciliogenesis and function in patient fibroblasts. EBioMedicine.

[10] Gagliardi G, Ben M'Barek K, Goureau O (2019). Photoreceptor cell replacement in macular degeneration and retinitis pigmentosa: a pluripotent stem cell-based approach. Prog Retin Eye Res.

[11] Garitaonandia I, Amir H, Boscolo FS, Wambua GK, Schultheisz HL, Sabatini K, Morey R, Waltz S, Wang YC, Tran H (2015). Increased risk of genetic and epigenetic instability in human embryonic stem cells associated with specific culture conditions. PLoS ONE.

[12] Giuliano CJ, Lin A, Girish V, Sheltzer JM (2019). Generating single cell-derived knockout clones in mammalian cells with CRISPR/Cas9. Curr Protoc Mol Biol.

[13] Guenou H, Nissan X, Larcher F, Feteira J, Lemaitre G, Saidani M, Del Rio M, Barrault CC, Bernard FX, Peschanski M (2009). Human embryonic stem-cell derivatives for full reconstruction of the pluristratified epidermis: a preclinical study. Lancet.

[14] Hsiao C, Palecek SP (2012). Microwell regulation of pluripotent stem cell self-renewal and differentiation. BioNanoScience.

[15] Jang J, Yoo JE, Lee JA, Lee DR, Kim JY, Huh YJ, Kim DS, Park CY, Hwang DY, Kim HS (2012). Disease-specific induced pluripotent stem cells: a platform for human disease modeling and drug discovery. Exp Mol Med.

[16] Jinek M, Chylinski K, Fonfara I, Hauer M, Doudna JA, Charpentier E (2012). A programmable dual-RNA-guided DNA endonuclease in adaptive bacterial immunity. Science.

[17] Koblan LW, Doman JL, Wilson C, Levy JM, Tay T, Newby GA, Maianti JP, Raguram A, Liu DR (2018). Improving cytidine and adenine base editors by expression optimization and ancestral reconstruction. Nat Biotechnol.

[18] Komor AC, Kim YB, Packer MS, Zuris JA, Liu DR (2016). Programmable editing of a target base in genomic DNA without double-stranded DNA cleavage. Nature.

[19] Li L, Wang BH, Wang S, Moalim-Nour L, Mohib K, Lohnes D, Wang L (2010). Individual cell movement, asymmetric colony expansion, rho-associated kinase, and E-cadherin impact the clonogenicity of human embryonic stem cells. Biophys J.

[20] Morizur L, Herardot E, Monville C, Ben MK (2020). Human pluripotent stem cells: a toolbox to understand and treat retinal degeneration. Mol Cell Neurosci.

[21] Peng R, Lin G, Li J (2016). Potential pitfalls of CRISPR/Cas9-mediated genome editing. FEBS J.

[22] Rowe RG, Daley GQ (2019). Induced pluripotent stem cells in disease modelling and drug discovery. Nat Rev Genet.

[23] Shivram H, Cress BF, Knott GJ, Doudna JA (2021). Controlling and enhancing CRISPR systems. Nat Chem Biol.

[24] Sondergaard JN, Geng K, Sommerauer C, Atanasoai I, Yin X, Kutter C (2020). Successful delivery of large-size CRISPR/Cas9 vectors in hard-to-transfect human cells using small plasmids. Commun Biol.

[25] Takahashi K, Tanabe K, Ohnuki M, Narita M, Ichisaka T, Tomoda K, Yamanaka S (2007). Induction of pluripotent stem cells from adult human fibroblasts by defined factors. Cell.

[26] Vallone VF, Telugu NS, Fischer I, Miller D, Schommer S, Diecke S, Stachelscheid H (2020). Methods for automated single cell isolation and sub-cloning of human pluripotent stem cells. Curr Protoc Stem Cell Biol.

[27] Watanabe K, Ueno M, Kamiya D, Nishiyama A, Matsumura M, Wataya T, Takahashi JB, Nishikawa S, Nishikawa S, Muguruma K (2007). A ROCK inhibitor permits survival of dissociated human embryonic stem cells. Nat Biotechnol.

[28] Yan J, Kang DD, Dong Y (2021). Harnessing lipid nanoparticles for efficient CRISPR delivery. Biomater Sci.

[29] Yang L, Soonpaa MH, Adler ED, Roepke TK, Kattman SJ, Kennedy M, Henckaerts E, Bonham K, Abbott GW, Linden RM (2008). Human cardiovascular progenitor cells develop from a KDR+ embryonic-stem-cell-derived population. Nature.

