# Supplementary figures and images for: Semi-automated optimized method to isolate CRISPR/Cas9 edited human pluripotent stem cell clones
Source: Stem Cell Res Ther. 2023 Apr 27;14:110. doi: 10.1186/s13287-023-03327-2 (PMC10142500; doi:10.1186/s13287-023-03327-2)

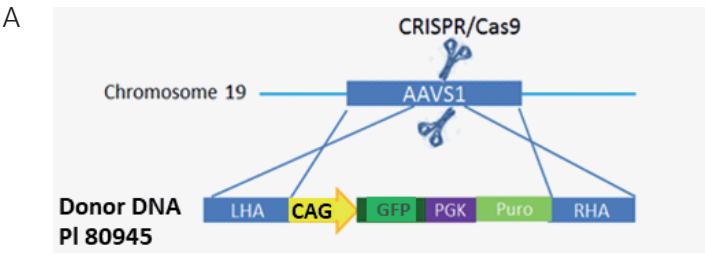

Isotypes      SSEA-4 / TRA1-81

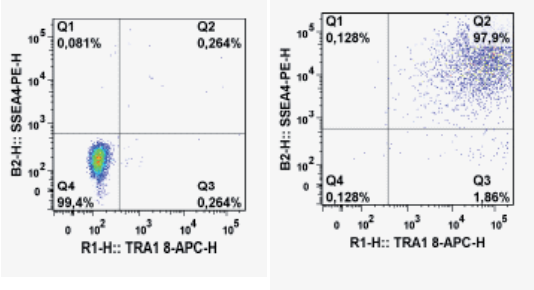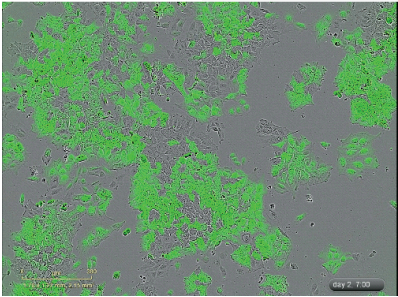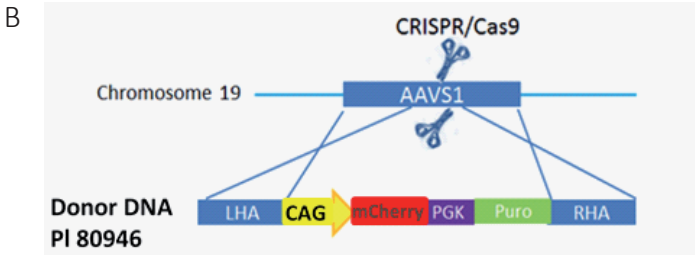

Isotypes      SSEA-4 / TRA1-81

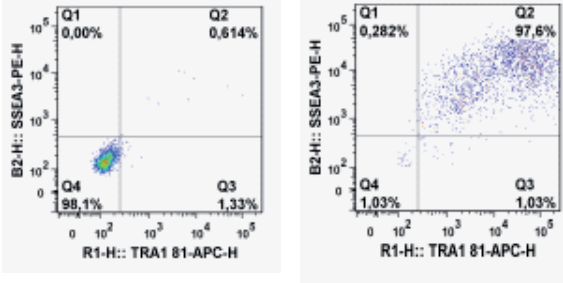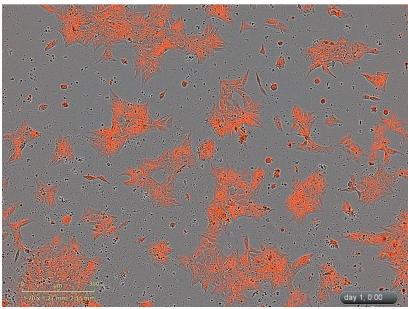

**C**

**D**

Supplement: Supplementary file 1 — Additional file 1: Fig. S1. Upper panel: Design of CRISPR/Cas9 edition into AAVS1 locus and donor DNA insertion to generate hiPSCs with constitutive expression of GFP and mCherry. Middle panel: Flow cytometry analysis of pluripotency markers SSEA-4 and TRA1-81 of edited hPSCs. Lower panel: images of edited hiPSCs with constitutive expression of GFP or mCherry. [file 13287_2023_3327_MOESM1_ESM.pdf]
