# Supplementary material for: Semi-automated optimized method to isolate CRISPR/Cas9 edited human pluripotent stem cell clones
Source: Stem Cell Res Ther. 2023 Apr 27;14:110. doi: 10.1186/s13287-023-03327-2 (PMC10142500; doi:10.1186/s13287-023-03327-2)
Supplement: Supplementary file 3 — Additional file 3: Fig. S2. A Single-nucleotide polymorphismcomparative analysis of parental cells and a representative edited single cell-derived hiPSC clone. B Table recapitulating the comparative analysis of original hiPSCs and clones derived with ALMS1 p.Glu192fs and ALMS1 p.Gln3613Ter mutations. C Number of clones analyzed for SNPs according to the CRISPR/Cas9 experiment. [file 13287_2023_3327_MOESM3_ESM.pdf]

A

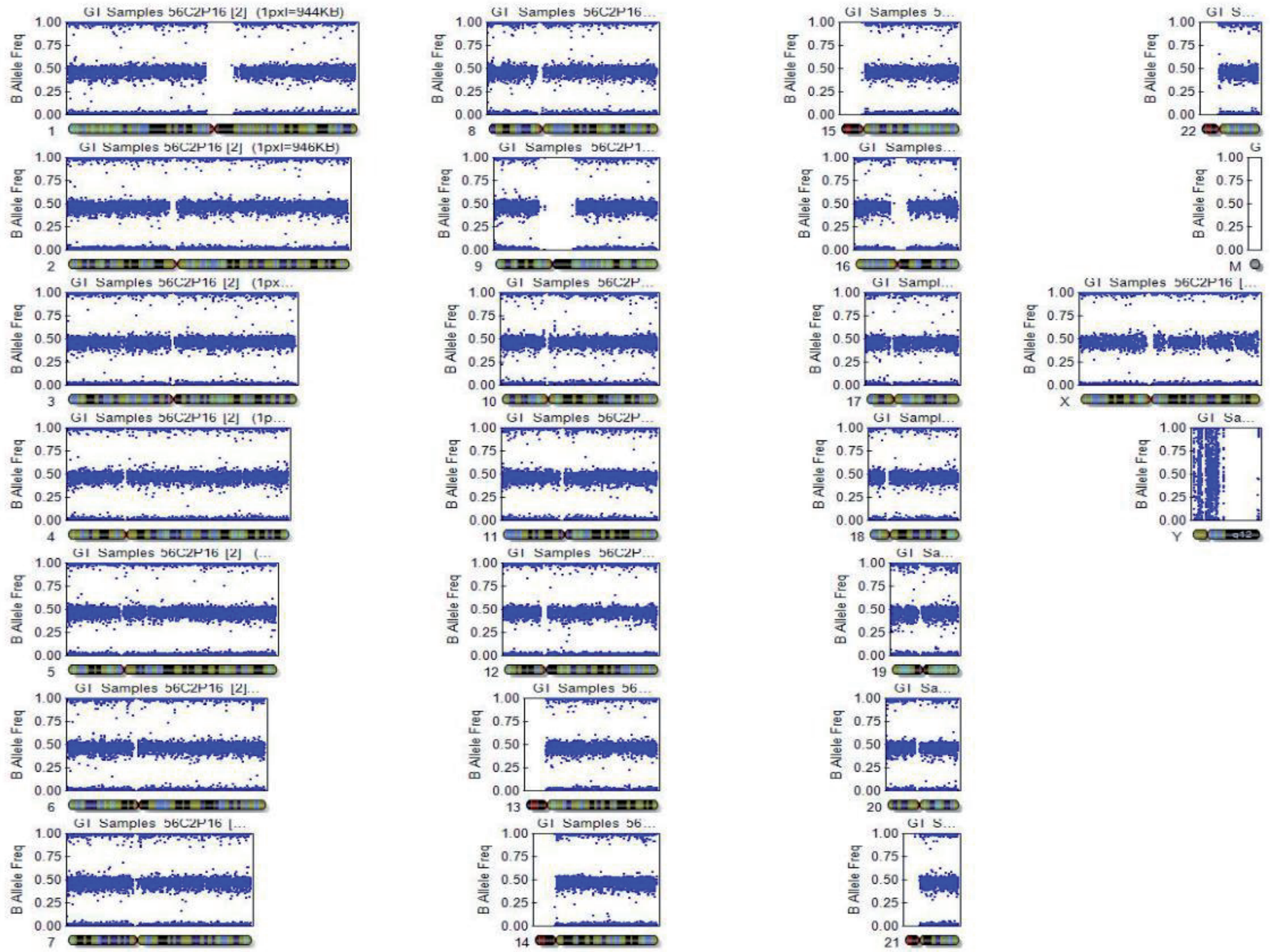

B

Wildtype	Chromosome 4					Chromosome 6					Chromosome 10				
	default	zone	start	end	default size	default	zone	start	end	default size	default	zone	start	end	default size
	duplication	q13.2	69.250.882	69.591.612	341 Kb	deletion	q12	66.998.589	67.072.560	74 Kb	duplication	10q11.22	47.543.322	47.703.869	160Kb
p.Glu192fs	Chromosome 4					Chromosome 6					Chromosome 10				
	similar to WT					similar to WT					similar to WT				
p.Gln3613Ter	Chromosome 4					Chromosome 6					Chromosome 10				
	similar to WT					similar to WT					similar to WT				

C

SNPs analysis	Number of clones analyzed	Number of clones conform to parental cell line
CRISPR KO edition	7	5
CRISPR base edition	3	2
